# Sterile Protection against *Plasmodium knowlesi* in Rhesus Monkeys from a Malaria Vaccine: Comparison of Heterologous Prime Boost Strategies

**DOI:** 10.1371/journal.pone.0006559

**Published:** 2009-08-10

**Authors:** George Jiang, Meng Shi, Solomon Conteh, Nancy Richie, Glenna Banania, Harini Geneshan, Anais Valencia, Priti Singh, Joao Aguiar, Keith Limbach, Kurt I. Kamrud, Jonathan Rayner, Jonathan Smith, Joseph T. Bruder, C. Richter King, Takafumi Tsuboi, Satoru Takeo, Yaeta Endo, Denise L. Doolan, Thomas L. Richie, Walter R. Weiss

**Affiliations:** 1 Naval Medical Research Center, Malaria Program, Silver Spring, Maryland, United States of America; 2 Henry M. Jackson Foundation, Rockville, Maryland, United States of America; 3 AlphaVax, Research Triangle Park, North Carolina, United States of America; 4 GenVec, Gaithersburg, Maryland, United States of America; 5 Cell-free Science and Technology Research Center, Ehime University, Matsuyama, Ehime, Japan; 6 Queensland Institute of Medical Research, Brisbane, Australia; University of Miami, United States of America

## Abstract

Using newer vaccine platforms which have been effective against malaria in rodent models, we tested five immunization regimens against *Plasmodium knowlesi* in rhesus monkeys. All vaccines included the same four P. knowlesi antigens: the pre-erythrocytic antigens CSP, SSP2, and erythrocytic antigens AMA1, MSP1. We used four vaccine platforms for prime or boost vaccinations: plasmids (DNA), alphavirus replicons (VRP), attenuated adenovirus serotype 5 (Ad), or attenuated poxvirus (Pox). These four platforms combined to produce five different prime/boost vaccine regimens: Pox alone, VRP/Pox, VRP/Ad, Ad/Pox, and DNA/Pox. Five rhesus monkeys were immunized with each regimen, and five Control monkeys received a mock vaccination. The time to complete vaccinations was 420 days. All monkeys were challenged twice with 100 P. knowlesi sporozoites given IV. The first challenge was given 12 days after the last vaccination, and the monkeys receiving the DNA/Pox vaccine were the best protected, with 3/5 monkeys sterilely protected and 1/5 monkeys that self-cured its parasitemia. There was no protection in monkeys that received Pox malaria vaccine alone without previous priming. The second sporozoite challenge was given 4 months after the first. All 4 monkeys that were protected in the first challenge developed malaria in the second challenge. DNA, VRP and Ad5 vaccines all primed monkeys for strong immune responses after the Pox boost. We discuss the high level but short duration of protection in this experiment and the possible benefits of the long interval between prime and boost.

## Introduction

Malaria infects over 200 million people annually and causes almost 1 million deaths [1]. An effective vaccine against malaria would be a valuable public health tool, complementing anti-malaria drugs, vector control and environmental modification. Despite intensive research no malaria vaccine is commercially yet available. The vaccine farthest along in field testing [2]is based on a single malaria antigen, and is not as effective as experimental radiation attenuated whole parasite vaccines [3]–[8]. When immune responses to the protective irradiated parasite vaccines are analyzed, no single target antigen has been identified that explains the full extent of host immunity[9]. This suggests that the protective vaccines work by the summation of many immune responses against multiple antigens on the parasites[9].

Our approach to vaccine development is to develop a multi-antigen malaria vaccine, mimicking the radiation attenuated whole parasite vaccines. However, until recently there has been no animal model allowing the efficacy testing of vaccines against the pre-erythrocytic stages of the human malaria parasite *P. falciparum*. One group in South America has shown that an Owl monkey can be reproducibly infected with sporozoites of *P. falciparum* [10]–[12]. However access to these protected primates is restricted making this model difficult to replicate elsewhere. While murine malaria models are invaluable for basic laboratory testing, they may not accurately predict human vaccine immunogenicity or efficacy. Furthermore there are no reliable immune correlates of protection for malaria vaccines, so immunogenicity studies without the results of malaria challenge are potentially misleading. Attempting to avoid these difficulties, we have chosen to test malaria vaccine strategies in the *P. knowlesi*/rhesus monkey system.

P. knowlesi is a natural infection of *Macaca fasicularis* (cynomolgus) monkeys[13], but also infects humans in South East Asia[14], [15]. P. knowlesi sporozoites are highly infectious for many primates including *M. mulatta* (rhesus) monkeys with 100 P. knowlesi sporozoites given iv reliably infecting rhesus monkeys in our facility. After the P. knowlesi sporozoite invades the hepatocyte, merozoites are released into the bloodstream 4–5 days later, comparable to the 5–6 day hepatic development of P. falciparum in humans. P. knowlesi takes only 24 hours to complete its growth cycle in the red blood cell, as compared to 48 hours for P. falciparum, and exponential growth of P. knowlesi often leads to parasitemias over 50% that can be fatal in rhesus. If the initial surge of parasites does not kill the host, P. knowlesi becomes a chronic low-grade infection with reproducible spikes in parasitemia due to antigenic variation[13], [16], similar to chronic P. falciparum infection in humans. P. knowlesi infection can be cured with chloroquine, and monkeys can be successfully re-infected with P. knowlesi sporozoites 4–6 times before significant blood stage immunity is evident ([13] and Weiss, unpublished data), which allows for repeat sporozoite challenges to assess the duration of vaccine protection.

Our goal in designing this experiment was to find a more potent malaria vaccine than the DNA/poxvirus heterologous combination which we have tested previously [17]–[19]. The vaccines we use combine four malaria antigens: the circumsporozoite protein (CSP), sporozoite surface protein 2 also called thrombospondin-related adhesion protein (SSP2 or TRAP), apical merozoite antigen-1 (AMA1) and merozoite surface protein 1 (MSP1). We refer to this four antigen combination as Pk4. Previously the best protection we have seen in rhesus monkeys was from a Pk 4 ‘prime-boost’ vaccine using DNA plasmids followed by recombinant poxvirus. In this experiment, 2/11 (18%) animals were sterilely protected, with an additional 7/11(63%) showing blood stage protection [18]. However, our studies of this vaccine have highlighted several limitations. First, there was little immune response detectable in the peripheral blood after the DNA vaccinations, which made us wonder if better priming before viral boost would be more efficacious. Secondly, protection by the vaccine waned quickly, and there was little efficacy to a second malaria sporozoite challenge given three months after the first challenge. Also, we did not have the reagents to measure immune responses to all four antigens in the Pk4 vaccine.

The present study uses the Pk4 antigens to compare priming with three different vaccine modalities before poxvirus (Pox) boost: DNA plasmids, recombinant adenovirus 5 (Ad5) [20], [21], and recombinant alphavirus-derived viral replicon particles (VRPs) [20], [21]. The DNA plasmids and poxviruses constructs used in this study are the same as used in our previous published work [17]. Our group has tested both VRP and Ad5 malaria vaccines in mice, and has found them to be as good as or better than DNA vaccines for priming before a poxvirus boost ([22] and Doolan unpublished data). Our goal was to evaluate these vaccine technologies in a primate malaria model where vaccine responses, host-parasite interactions and protective efficacy may be better predictors of results in humans than can be achieved with rodent malaria models. We also developed reagents to test immune responses to all 4 P. knowlesi vaccine antigens in order to study their association with protection.

## Materials and Methods

### Animals

Rhesus monkeys (*Macaca mulatta*) descended from Chinese stock were used for this experiment. Animals were obtained by and housed at the Walter Reed Army Institute of Research/Naval Medical Research Center (WRAIR/NMRC), Silver Spring, MD. Animals were selected to be in general good health, and to have no history of prior exposure to malaria. Prior to selection for the studies, serum specimens from all animals were tested in IFAT assays against P. knowlesi sporozoites and P. knowlesi infected red cells, and all animals with positive serum titers at dilutions of 1∶80 or higher were excluded. The experiment was conducted according to *Guide for the Care and Use of Laboratory Animals* 1996. The experiment required 6 groups of 5 monkeys each (see Table 1). The 30 selected monkeys were first stratified by age, sex, and weight and then randomly assigned to groups. This resulted in the 6 groups being closely matched, with mean age 6.4 years (SD 0.2) and mean weight 5.2 kg (SD 0.2). There were 2 females and 3 males in each group.

**Table 1:** Immunization regimens.

Group^a^	Vaccinations^b^	
	wk 0	wk 4	wk 16	wk 55	wk 60	wk 62
**Control**	-	-	-	-	pPox^f^	challenge^h^
**Pox**	-	-	-	-	Pox^g^	challenge
**VRP/Pox**	VRP^c^	VRP	VRP	-	Pox	challenge
**VRP/Ad**	VRP	VRP	VRP	-	Ad5	challenge
**Ad/Pox**	-	-	-	Ad5^d^	Pox	challenge
**DNA/Pox**	Plasmid^e^	Plasmid	Plasmid	-	Pox	challenge

a Rhesus monkeys 5 animals per group.

b Vaccines are mixtures of vectors expressing the individual antigens PkCSP, PkAMA1, PkSSP2, and PkMSP1.

c Recombinant VRP, 5×10^7^ IU/dose each antigen.

d Recombinant Ad5 vectors, 2.5×10^10^ particles each antigen.

e Recombinant plasmid vaccine 1 mg/dose each antigen.

f Parental pox virus without antigen inserts, 8×10^8^ pfu total.

g Recombinant pox virus, 2×10^8^ pfu/dose each antigen.

h Challenge with 100 Pk sporozoites iv 12 days after last vaccination.

### Ethics Statement

Animal use in this study was approved by the WRAIR/NMRC Institutional Animal Care and Use Committee. The WRAIR/NMRC animal facility is AAALAC accredited and animals are housed and cared for according to its guidelines. In this study the major risk to the animals was from the malaria infection. Harm from malaria infection was minimized by treating with anti-malarial drugs at a parasitemia level low enough to prevent serious illness.

### DNA plasmid vaccines

The DNA plasmid vaccines encoding Pk4 genes have been previously described [17]. Briefly, DNA sequences encoding the full-length genes from the P. knowlesi H strain of CSP, SSP2, and AMA-1 and the 42 kD C terminal fragment of MSP-1 were cloned into the VR1020 mammalian expression vector (Vical Inc, San Diego CA). This vector contains a CMV promoter, and a TPA signal sequence. Each gene was cloned into a separate plasmid. Recombinant DNA plasmids were produced by Vical, Inc and contained less that 0.6 EU of endotoxin per mg and were at least 80% super coiled. Plasmids were diluted in PBS pH 7.2 prior to injection.

### Viral vectors

The same sequences of the Pk4 genes were cloned into 3 different viral vectors: VRP, Ad5, and Pox. Each P. knowlesi antigen was cloned into separate virus vector.

The Pox vaccines encoding P. knowlesi genes have been previously described [17], [18]. Briefly, the same four P. knowlesi DNA sequences, which were used to construct the P. knowlesi DNA plasmids, were cloned into the COPAK poxvirus immunization vector (Virogenetics, Troy, N.Y). COPAK is derived from the Copenhagen strain of vaccinia virus. The recombinant alphavirus derived VRP particles for the Pk4 vaccine were constructed and produced by AlphaVax, Inc (Research Triangle Park, NC), and the recombinant attenuated Ad 5 for the Pk4 antigens were produced by the GenVec, Inc (Gaithersburg, MD).

### Immunization regimens

Five Pk4 malaria vaccine regimens were compared to a mock control vaccine in this experiment (Table 1). At the time of each injection, the four antigen vaccines (either DNA or viruses) were mixed and then given im in the right quadriceps muscle in a total volume of 1 ml. DNA injections were given by a needle-free injection system Biojector 2000 (Bioject, Inc, Tualatin, OR), while all other injections were with #20 gauge needle and syringe. As seen in Table 1, groups received either no priming injections, or were primed with DNA plasmids, VRPs, or Ad5. DNA priming injections contained 1 mg of each of the four Pk4 plasmids, and were given at weeks 0, 4, and 16. VRP priming injections contained 5×10^7^ infectious units (IU) encoding each Pk4 antigen, and were also given at weeks 0, 4, and 16. The Ad5 priming injection contained 2.5×10^10^ particles encoding each Pk4 antigen and was given at week 55.

All monkeys were boosted at week 60. The Control group was given 8×10^8^ pfu of parental COPAK virus lacking a transgene insert. The four groups receiving Pox vaccine were given a mix of 2×10^8^ pfu of each of four COPAK viruses encoding one of the four Pk4 antigens. The one group boosted with Ad5 received a mix of 2.5×10^10^ particles of each of the four Ad5 viruses encoding one of the four Pk4 antigens (same dose as the Ad5 prime).

### Malaria sporozoite challenges and parasitemia measurement

The first P. knowlesi sporozoite challenge was given on day 12 after viral boost (week 62). Our initial plan was to challenge 2–4 weeks after viral boost as we had done in our previous studies [17]–[19]. However, the challenge was done two days early when it appeared that this was the best date to obtain infectious sporozoites from our mosquitoes. P. knowlesi H strain sporozoites were grown in *Anopheles dirus* mosquitoes. Sporozoites were harvested 14 days after mosquitoes had fed on an infected rhesus monkey. Harvesting was by the Ozaki method. Sporozoites were diluted in E199 medium with 5% normal rhesus serum and counted with a hemocytometer. 100 sporozoites in a total volume of 1 ml were injected IV. A random challenge order was used for monkeys from different groups, with the exception that the first and last monkeys challenged were from the Control group. The challenge took place over the course of four hours.

Beginning 6 days after sporozoite challenge, each day at 1 PM blood was taken by ear prick. P. knowlesi infections are highly synchronized in the blood. Before noon parasites are schizonts, up to half of which may adhere to blood vessels making counts of circulating parasites inaccurate. With low levels of parasitemia, most schizonts rupture around mid-day to produce a new crop of ring forms. Taking blood samples at 1pm avoids underestimating parasite load during the early days of infection. At higher parasitemia levels, schizont rupture is often delayed several hours. If many schizonts are present in the 1 PM specimen, blood smears were repeated later in the day to get accurate parasite counts. Blood was prepared for thin and thick malaria smears using Giemsa stain at pH 7.01 using standard methods [23] For thin smears, 20,000 red cells were examined. For thick smears, 0.025 µl of blood was examined. These data was used to calculate the percent infected red blood cells. Animals were followed for 40 days after challenge. To prevent death of animals, when parasitemias exceeded 2% monkeys were treated by IM injection of chloroquine hydrochloride 20 mg/kg on day 1 and 10 mg/kg on days 3 and 4. Forty days after the first sporozoite challenge all previously untreated monkeys received chloroquine to eliminate any possible undetected malaria infections prior to rechallenge.

The second P. knowlesi sporozoite challenge was given four months after the first sporozoite challenge using the same procedures for infection and follow-up of parasitemias.

#### Blinding for antibody and T cell assays

Operators conducting the antibody and T cell assays were not aware of the vaccination group or the parasitemia status of animals when they performed the assays. When T cell assays had to be run in batches, a study investigator who was not involved in the in vitro testing selected the samples, such that animals from all groups were included in every run to exclude inter-group bias.

### Antibody ELISA assay

Plasma sample was tested by ELISA for IgG titer using each of the four P. knowlesi antigens used in the immunization studies as capture antigens. Capture antigen for P. knowlesi CSP was a synthetic peptide of 36 amino acids representing 3 copies of the 12 aa repeat motif GDGANAGQPQAQ. Capture antigen for the other three proteins consisted of full length P. knowlesi SSP2, full length P. knowlesi AMA-1 ectodomain and the P. knowlesi MSP-1 42 kD fragment, respectively, each produced by in vitro synthesis using the Rapid Translation System RTS 500 E. coli HY kit (Roche Diagnostics Corporation, Indianapolis, IN). These capture antigens were used at concentrations of 1 to 4 micrograms per ml in PBS pH 7.2 in Immulon II 96 well plates (Dynex Technologies Inc., Chantilly, VA). Plates were blocked with 5% milk powder in PBS for 2 hours. Plasma samples were diluted in 3% non-fat dry milk in PBS and incubated in plates at room temperature for 4–18 hours. Peroxidase-labeled goat anti-human IgG (H+L) (Kierkegard Perry Laboratories, Gaithersburg MD) at a 1∶10,000 dilution in 3% non-fat dry milk was added for 1 hour, and subtrate was ABTS (Kierkegard Perry Laboratories). OD was read using a SPECTRA MAX 190 ELISA reader (Molecular Devices Corp., Sunnyvale, CA). Endpoint titer for each sample was the highest plasma dilution at which the OD was equal or greater than 3-fold the value of plasma from naïve monkeys.

### Immunofluroescence Antibody titers (IFAT) against whole parasite preparations

For each animal, plasma from five days before the first sporozoite challenge was evaluated in IFAT against both P. knowlesi air dried sporozoites and P. knowlesi infected red blood cells as previously described [24]. Results were the last dilution of plasma at which fluorescence could be seen.

### Antigens for in vitro studies of T cells

For in vitro T cell studies of the four P. knowlesi strain antigens, we restimulated cells using synthetic peptides for the CSP and AMA1 antigens, and recombinant proteins for the SSP2 and MSP1 antigens. For all studies, negative control wells were run with medium alone, and positive control wells were run with concanavalin A. Synthetic peptides for the CSP and AMA1 antigens were produced by Pepscan (Lelystad, The Netherlands). Each peptide was 15 amino acids long with 10 amino acids overlapping the adjacent peptide, and the peptide series covered the entire length of each P. knowlesi protein. The CSP pool contained 42 peptides and the AMA1 pool contained 104 peptides. The final concentration of each individual peptide in the pool was 2.5 µg/ml for the CSP and 1.2 µg/ml AMA1. These concentrations were selected based on our previous studies, and testing with samples from a small number of positive and negative control samples.

The recombinant P. knowlesi SSP2 and MSP1 (42 kD) proteins used for in vitro T cell restimulation were generated using an in vitro wheat-germ cell free expression system. This protein expression method has been described in detail[25]–[27]. Briefly, transcription of mRNA was achieved using SP6 RNA polymerase (Promega, Madison, WI). The reaction mixture resulting from transcription is then directly used as mRNA source in the translation step. Proteins were translated using a cell-free bilayer system [26], where the translation reaction is separated from translational substrate buffer by carefully overlaying in a 6-well multi-well plate. Then the plate was incubated at 26°C for overnight. Proteins from the reaction were bound to a glutathione sepharose 4B column (GE Healthcare Bio-Sciences, Piscataway, NJ), washed with PBS, and then the column was treated with TEV protease (Invitrogen, 60 U/column) at 30°C for 3 hrs. Proteins were eluted with PBS and fractions were analyzed by 12.5% SDS-polyacrylamide gel stained with CBB. Aliquots were stored at −80°C and proteins were used at a final concentration of 5 µg/ml in in vitro T cell studies.

### IFN-γ ELISPOT assay

For in vitro T cell studies, PBMC were isolated from peripheral blood by centrifugation over ficoll and preserved in liquid nitrogen. Cells from all time points for each animal were run on the same day to facilitate comparisons.

The assay for rhesus IFN-γ was modified from our previous method [28], [29]. In brief, PVDF-96 well plates (Millipore Corporation, Bedford, MA) were coated with anti-human IFN-γ (clone GZ-4, Bender Med Systems, Burlingame, CA) incubated overnight at 4°C, blocked and washed. Cryopreserved PBMC were rested overnight after thawing, and 2×10^5^ cells added per well. Quadruplicate cells were restimulated with one of the four P. knowlesi H strain antigens (as described above), or controls. ELISPOT plates were incubated for 18 hrs at 37°C in an atmosphere of 5% CO_2_. The IFN-γ spot-forming cells (SFCs) were counted using an AID ELISPOT reader (Cell Technology, Inc, Columbia, MD, USA). Responses are presented as the mean number of net SFCs per million cells in stimulated wells minus the mean number of spots in medium controls. The major differences from our previous methods are the resting of the PBMC after thawing, the use of PVDF instead of MAIP plates, and counting of spots with the AID ELISPOT reader.

### Intracellular cytokine staining and flow cytometric studies

All reagents for the intracellular cytokine staining were purchased from BD Bioscience (San Jose, CA) unless otherwise mentioned. A total 0.5-1×10^6^ cryopreserved PBMC were plated per well in U-bottomed 96-well plates, with 1 µg/ml anti-human CD28 (Clone CD28.2) and 1 µg/ml anti-human CD49d (Clone 9F10) antibodies with or without antigen. Malaria antigens and positive and negative control antigens were the same as for the ELISPOT studies. Brefeldin A was added at 10 µg/ml at 2 hrs after initial incubation, and plates then incubated an additional 14-hrs at 37°C in an atmosphere of 5% CO_2_. The cells were stained with one or more of the following antibodies: CD3-PE-Cy7, CD4-Alex430, and CD8-APC-Cy7. After the surface staining, cells were permeabilized in 100 µl CytoFix/Cytoperm buffer for 20 min, and then stained with anti- IFN-γ- FITC (Clone B27), and anti-IL2-APC(Clone MQ1-17H12), for 45 min on ice in the dark. The stained samples were analyzed using the LSR-II flow cytometer (Becton Dickinson Immunocytometry Systems, San Jose, CA). The expression level of intracellular cytokines was presented as the percentage of stained cells in gated cell populations minus background responses in the absence of antigen. The non-specific background was generally between 0.001–0.05%.

### Statistical analyses

Parasitemia outcomes were analyzed using Kaplan-Meier survival curves and the Log rank test has been used to compare survival curve for two or more groups. Immunogenicity of vaccine groups was analyzed using ANOVA and Tukey's Adjusted Significant Difference Test. We used the Cox Proportional Hazard model to analyze effects of immune responses on protection against malaria.

## Results

### Effect of vaccinations on parasitemias

**Figure 1:**
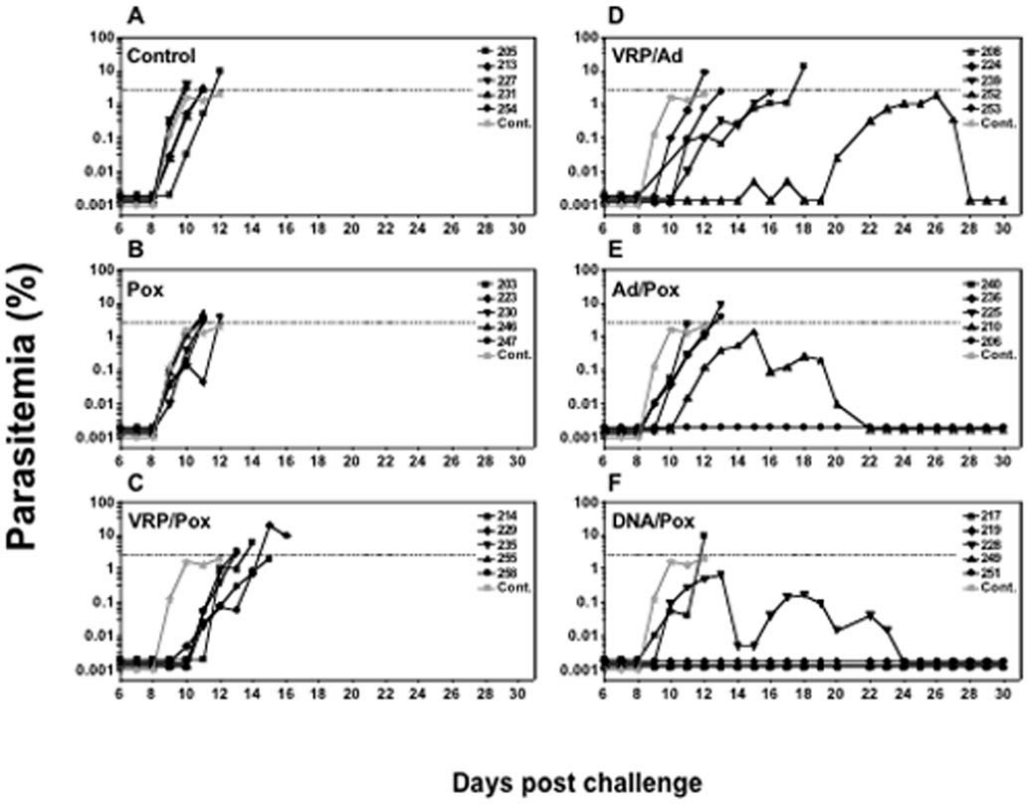
Daily parasitemias from individual monkeys after sporozoite challenge. Panel A, Control group: average parasitemia levels of 5 individual animals was presented as a thick grey line (Cont.) and is included in all 6 panels for comparison; B, Pox group; C, VRP/Pox group; D,VRP/Ad group; E, Ad/Pox group; F, DNA/Pox group; The dotted line in each panel shows the parasitemia level 2% at which we treated animals with anti-malaria drug. One monkey (206) in Panel E, and 3 monkeys (219, 249 and 251) in Panel F that had no detectable parasitemia are shown as horizontal lines.

Figure 1 shows the parasitemia curves for all monkeys after the first sporozoite challenge. Each of the panels shows animals that received a different vaccine. In the Control group (Figure 1A), the first blood stage parasites were detected between days 8 to 10 (mean 8.6 days) and animals required drug treatment for parasitemia exceeding 2% between days 10 to 12 (mean 10.8 days). This is consistent with our previous studies using the 100 P. knowlesi sporozoite challenge in rhesus monkeys [17]–[19]. Animals receiving only recombinant Pk4 Pox vaccine 12 days before challenge (Figure 1B) had parasitemias similar to the Control group, with no protection against sporozoite or blood stage parasites.

Compared with the Control groups, monkeys receiving the VRP/Pox regimen (Figure 1C) had a 2.4 day delay to first parasitemia (mean 11 days) and a 3.2 day delay to parasitemia>2% (mean 14 days). Similar delays to the first parasites being detected were seen in the VRP/Ad group (Figure 1D), with monkey #252 having an unusual pattern of infection. This animal did not have detectable parasites in thick or thin malaria blood films until days 15–19, when single parasites were observed intermittently. Then, starting on day 20, parasitemia rose steadily for one week, peaking at 1%, followed by a decline that occurred in the absence of drug treatment. We suspect this animal was parasitemic at a level below detection prior to day 15, and then had a increase in parasites due to antigenic variation [30], a pattern of recrudescence well known in P. knowlesi infections and one we have observed previously in our own studies [17], [18]. The control of parasitemia below lethal levels without need of drugs we term ‘self-cure,’ although it is likely that blood stage parasites remain at very low levels after they fall below the threshold detectable by microscopy.

Figure 1E shows results from the Ad/Pox vaccine group. For three of the animals in this group there was a modest 0.9 day delay in day to first parasitemia (mean 9.5 days) and 1.5 day delay in the day>2% level (mean 12.3 days), relative to Controls. A fourth animal reached 1% parasitemia and then self-cured. The fifth animal never developed detectable parasitemia during the 40 days of follow up. We think that this animal was sterilely protected by the Ad/Pox vaccine and never had P. knowlesi parasites exit the liver and infect red blood cells, because there were no later spikes of recrudescent parasitemia (as observed in the self-cure monkey #252 from Panel D) during the 40 days of follow-up.

Figure 1F shows the parasitemias of the DNA/Pox vaccine group. Three of five monkeys were sterilely protected, with 2 monkeys showing a 0.9 day delay in first day of parasitemia (mean 9.5 days). One of the two infected animals controlled its initial parasitemia at 1% and then self-cured, while the other exceeded 2% parasitemia on day 12. We have never previously sterilely protected such a high proportion of animals given any Pk4 vaccine. We discuss possible reasons for this high level of protection below.

**Figure 2:**
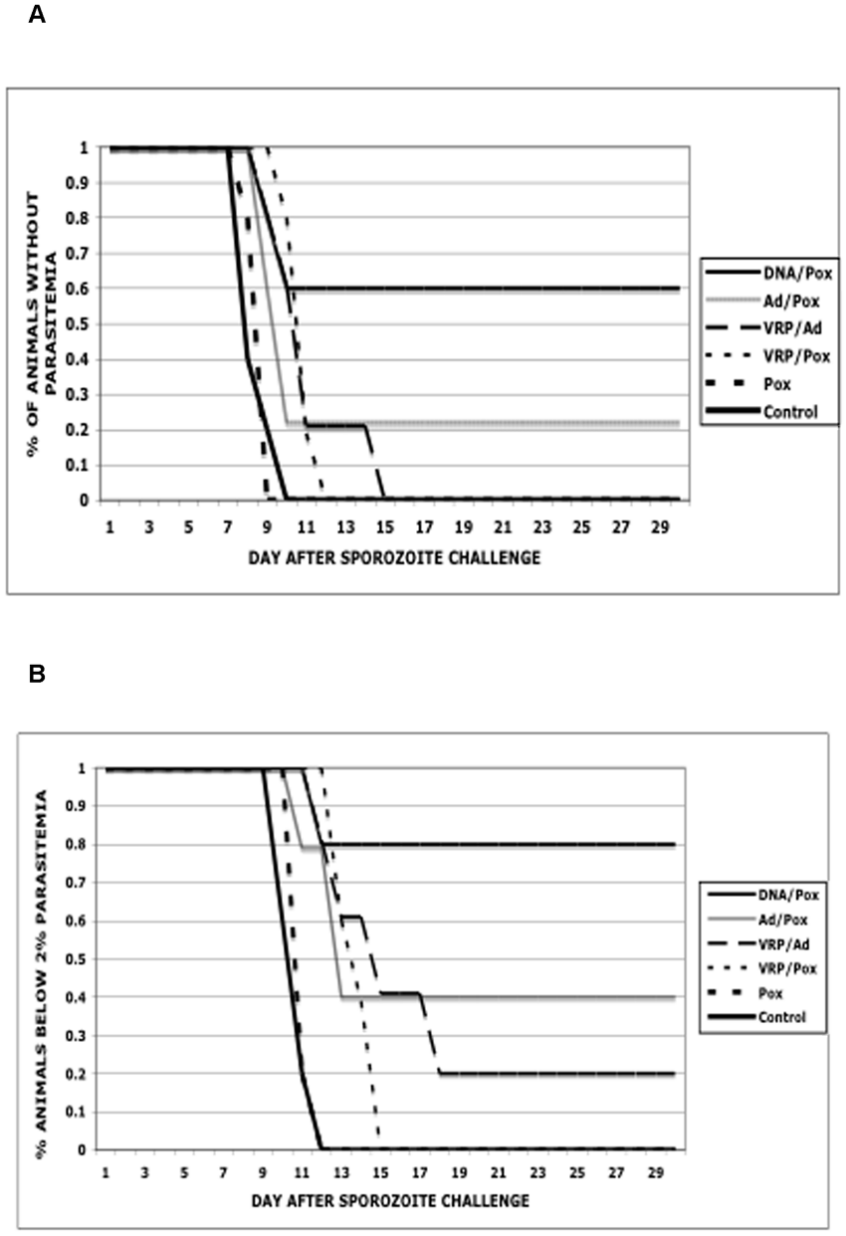
Kaplan-Meyer curves showing parasitemia endpoints for the six experimental groups. Panel A. shows the percentage of animals in each vaccine group without parasites detected in blood. Panel B. shows the percentage of animals with parasitemia below 2%. X axis shows day since sporozoite challenge. In each panel the DNA/Pox group shows the highest level of protection.

Fig. 2a shows Kaplan-Meyer curves of the percentage of animals in each group having parasites detected in the blood by day after challenge. Figure 2b is a similar graph showing the percentage of each group exceeding 2% parasitemia by day after challenge. The DNA/Pox group is the only vaccine group that had any endpoints statistically different from the Control group (p = 0.06 and 0.02 for day of first parasitemia and>2% parasitemia respectively, Log-rank Test). The other vaccine groups appear less protective than the DNA/Pox vaccine but differences do not reach statistical significance.

### Pk4 vaccine effects against specific stages of the malaria lifecycle

Because the Pk4 vaccine contains antigens that are expressed in sporozoites, liver stages, and blood stages of malaria, it is difficult to assign protective roles to particular vaccine components. CSP and SSP2 are found on sporozoites and in early hepatic stages. AMA1 and MSP1, which are expressed during late hepatic stages and merozoites, could contribute to protection at both the hepatic and blood stages of infection. In addition, there is evidence that AMA1 is present in sporozoite[31]. The time to first detection of parasites in the blood could be increased by vaccine effects at several points in the life cycle: by inhibition of sporozoite invasion of liver cells, by killing of infected hepatocytes or prolongation of hepatic parasite maturation, or by inhibition of parasite replication in red cells. Because a slowing of parasite growth provides more time for induction of immune responses to the blood stages of the parasite, delays in the early phases of infection could also affect peak parasitemias. Thus prolongation of either endpoint (time to first parasite detected in the blood or time to reach>2% parasitemia) may indicate a mixture of stage-specific and antigen-specific effects.

**Figure 3:**
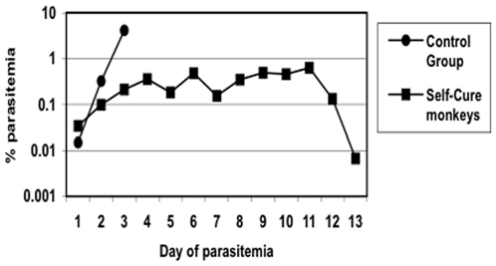
Mean parasitemias of the 5 Control monkeys compared to the 3 monkeys from vaccine groups which contained their parasitemia below 2%. The X axis is normalized so day 1 is the first day parasites were detected in the blood for each animal. Monkeys which controlled their parasitemias had slower growth rates.

However, two outcomes have clear relationships to protection against specific stages of the parasite life cycle. The first is sterile protection. On the assumption that the release of any parasites from the liver will eventually lead to a patent infection (such as animal 252, Panel D, Figure 1), we believe that the four animals that never had parasitemia provide evidence for complete protection against sporozoites and liver stages of the parasite. The second outcome with a straightforward interpretation is self-cure. Three animals became infected but limited their parasitemia without need for drug treatment, indicating an effective immune response against the blood stages of the parasite. Even prior to their decline in parasite counts, the self-cure animals showed a slower rate of growth than Controls (Figure 3). Between days 1–2 and 2–3 of parasitemia, the mean rate of increase for the 5 Control animals was 0.03% and 0.37% per day, a nearly exponential progression. For the 3 self-cure monkeys, the mean rate of increase between days 1–2, 2–3, and 3–4 was 0.07%, 0.11%, and 0.15%, a more constant rate of growth. We believe that immune responses to blood stage antigens must have caused these slower growth rates.

### Immunogenicity of different vaccine regimens

**Figure 4:**
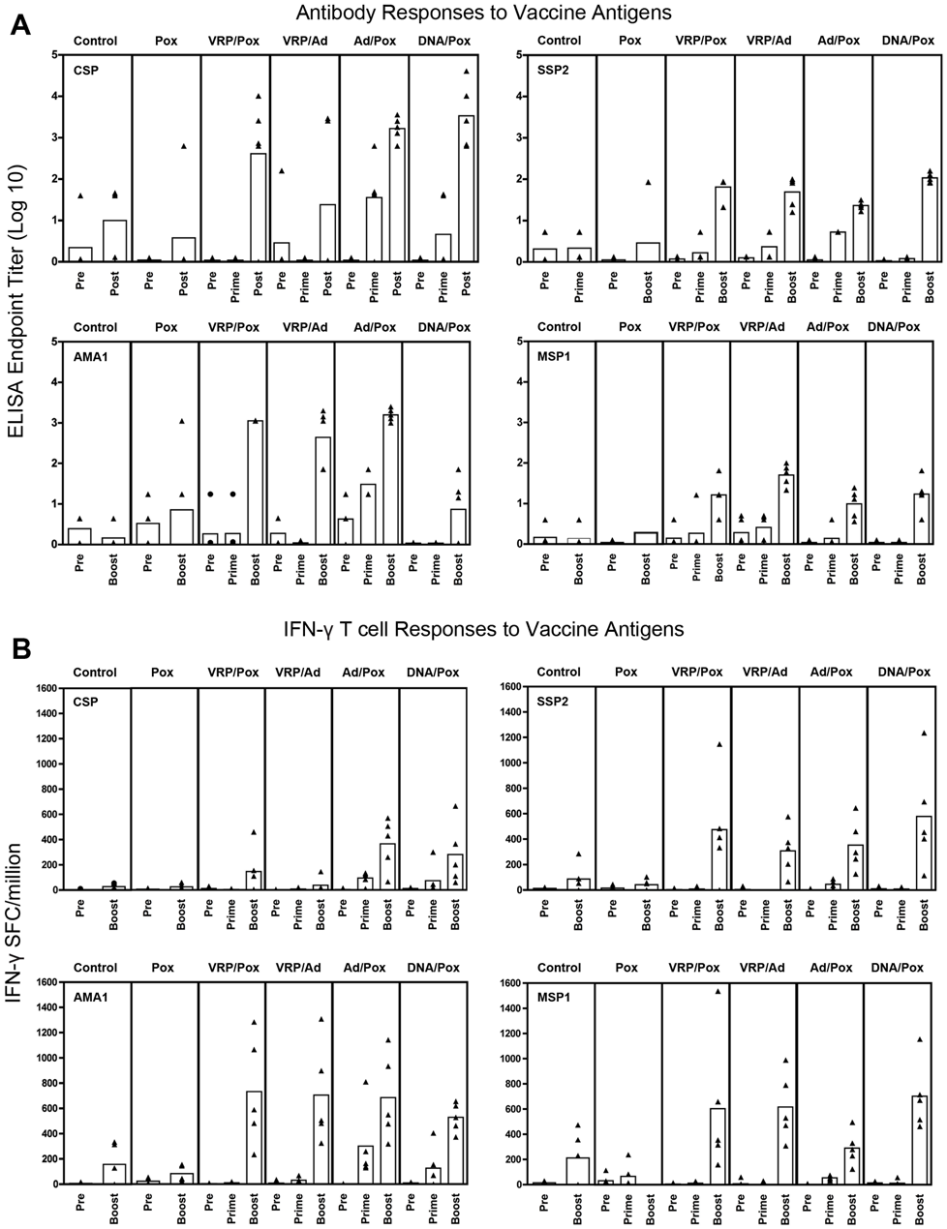
Immune responses induced by vaccinations against the four vaccine antigens. Plasma samples were assayed by ELISA (Panel A), and PBMCs were assayed -forming cells by Elispot (Panel B). Data is presented for 3 timeγfor IFN points: ‘Pre’ = pre-immunization; ‘Prime’ = 3 weeks after the last priming immunization; and ‘Boost’ = 7 days after viral boost immunization which was 5 days before challenge. Mean of 5 animals in each group was presented as a rectangle. Data for individual animals is presented as triangles. Immune responses in the Pox only group were lower than in all groups receiving prime/boost vaccines.

We measured circulating antibody and T cell responses to each of the four P. knowlesi antigens in the vaccine. Antibody measures included ELISA against each of the four vaccine antigens, and IFAT against whole fixed sporozoites and infected red cells. ELISA data is shown in Figures 4a. IFAT data is similar to ELISA data, with high titers to CSP or SSP2 giving high IFAT titers against sporozoites, and high ELISA titers to AMA1 or MSP1 giving high IFAT titers against infected red cells (data not shown). T cell responses were measured by IFN-γ ELISPOT assay and flow cytometric analysis of intracellular IFN-γ and IL-2 production. Data from the on IFN-γ ELISPOT on PBMCs are shown in 4b. Data from the flow cytometric studies were comparable to the IFN-γ ELISPOT (data not shown).

No significant immune responses were detected in samples from pre-vaccination samples. Three weeks after the last of three vaccinations with either Pk4 VRPs or Pk4 DNA plasmids, we detected no statistically significant immune responses to any of the four antigens by ELISA or ELISPOT. In contrast, three weeks after a single dose of Pk4 Ad5 (Figure 4a), there were significant increases in antibody responses to three of four antigens CSP, SSP2, and MSP1 p<0.05). The Ad5 vaccine also induced measurable ELISPOT responses to each of the four antigens in some animals(Figure 4b), although the group differences were not statistically significant.

All monkeys received a viral ‘booster’ vaccination at week 60 with either a control poxvirus (Control group), the Pk4 Ad5 viruses (VRP/Ad group), or the Pk4 poxviruses (four remaining groups). Blood was taken seven days later for measurement of immune responses and sporozoite challenge occurred 5 days after this blood sampling.

In the samples taken 7 days after the final vaccination, there were significant differences between the experimental groups in ELISA and ELISPOT responses to each of the four vaccine antigens (analysis by ANOVA, results not shown). We then compared immune responses with the Control group by T test using Tukey's Adjustment for multiple comparisons (Table 2). The group vaccinated with control Pox or Pk4 Pox (unprimed) seven days previously had no immune responses significantly different from Controls. In contrast, all prime/boost vaccine groups developed immune responses to some or all vaccine antigens that were statistically different from Controls, but there were no statistically significant differences between any prime/boost vaccine groups. Of note, the Ad/Pox and DNA/Pox vaccines were the only ones which induced significant ELISPOT responses to CSP, and these two vaccine groups were the only two which contained sterilely protected animals. The DNA/Pox group with 3/5 animals sterilely protected was the only vaccine group which produced statistically significant antibody responses to CSP.

**Table 2:** Immune responses of vaccine groups prior to challenge.

		Pox	VRP/Pox	VRP/Ad	Ad/Pox	DNA/Pox
**ELISPOT**	**CSP**				**+**	**+**
	**SSP2**			**+**	**+**	**+**
	**AMA1**		**+**	**+**	**+**	**+**
	**MSP1**		**+**	**+**	**+**	**+**
**ELISA**	**CSP**					**+**
	**SSP2**					
	**AMA1**		**+**	**+**	**+**	
	**MSP1**			**+**		

The five vaccine groups compared with the Control group for immune responses to each vaccine antigen. Analysis used Student's T test with Tukey's Adjustment for multiple comparisons. Crosses (**+**) indicate that the comparison with the Control group is statistically significant (p<0.05).

### Association between immune responses and protection against sporozoite challenge

We were interested to know if the magnitude of any immune response was associated with protection against malaria independent of which vaccine the animal received. We approached this question in two ways. First we analyzed immune responses of all 30 monkeys with respect to the two protective endpoints, ‘day of first parasitemia’ or ‘day>2% parasitemia’. As discussed previously, we believe that immune responses to both pre-erythrocytic and erythrocytic stage antigens could contribute to any protective effect identified by these two endpoints. In the second approach, we focused on the four sterilely protected monkeys (sterile protection reflecting immune responses targeting pre-erythrocytic stages) and the three monkeys that self-cured their parasites (self-cure reflecting immune responses targeting blood stages), comparing immune responses in these protected animals to the other monkeys in the same vaccine groups.

To analyze the relationship between immune responses of all 30 monkeys and protection we used Cox Proportional Hazard analysis. Table 3 shows that considered one at a time many immune responses to vaccine antigens were significantly associated with protection. All ELISPOT responses except for CSP had important effects on both day to first parasite and day>2% parasitemia. We have found this same lack of correlation of ELISPOT responses of PBMC to CSP in previous P. knowlesi vaccine studies [19]. For the ELISA data, both SSP2 and MSP1 responses had a significant effect on both protective endpoints. However, when we fit models using the immune responses to the four vaccine antigens simultaneously, neither ELISA nor ELISPOT responses to any one vaccine antigen were significantly correlated with either protective endpoint (data not shown).

**Table 3:** Cox Proportional Hazards Analysis of immune responses and parasitemia.

		Day of 1^st^ parasitemia	Day>2% parasitemia
**ELISPOT**	**CSP**		
	**AMA1**	**+**	**+**
	**SSP2**	**+**	**+**
	**MSP1**	**+**	**+**
**ELISA**	**CSP**		
	**AMA1**		
	**SSP2**	**+**	**+**
	**MSP1**	**+**	**+**

Crosses (**+**) show statistically significant effects on protective endpoints when immune responses are analysed separately. When responses to all antigens are analysed sim-ultaneously no single immune response is statistically associated with either protective endpoint.

In a second set of analyses, we focused on the immune responses in the sterilely protected and self-cure animals. We compared the immune responses to all four vaccine antigens of these protected animals with those of the other non-protected monkeys in the same vaccine groups. Comparing responses of the four sterilely protected monkeys with the six unprotected animals in the DNA/Pox and Ad/Pox groups, only the MSP1 ELISA and ELISPOT showed a trend toward higher values in the sterilely protected monkeys but this was not statistically significant (data not shown). There were also no differences in immune responses between the three self-cure monkeys, the four sterilely protected monkeys and the eight unprotected monkeys in the DNA/Pox, Ad/Pox, and VRP/Pox groups (data not shown). Thus, neither analysis allows a clear dissection of the protective roles of the different vaccine antigens. This is possibly explained by the fact that all immune responses were highly correlated with each other, so statistical separation of effects was not possible.

### Intracellular cytokine expression of CD4 and CD8 cells

To further understand the T cell responses to the four vaccine antigens, we used flow cytometry to study the CD4 and CD8 phenotype of T cells responding after in vitro restimulation using both IFN-γ and IL-2 production as measures of immune response. No increase in CD8+ T cell responses was detected for any of the four vaccine antigens (data not shown). In contrast, CD4+ T cells were detected producing IFN-γ, IL-2 or both cytokines together in a pattern similar to that seen in the IFN-γ ELISPOT assay (data not shown). We conclude that the ELISPOT responses from PBMC are primarily from CD4+ T cells, which has also been the case in our previous studies of the Pk4 vaccine in rhesus monkeys [28].

### Protection against a second sporozoite challenge

**Figure 5:**
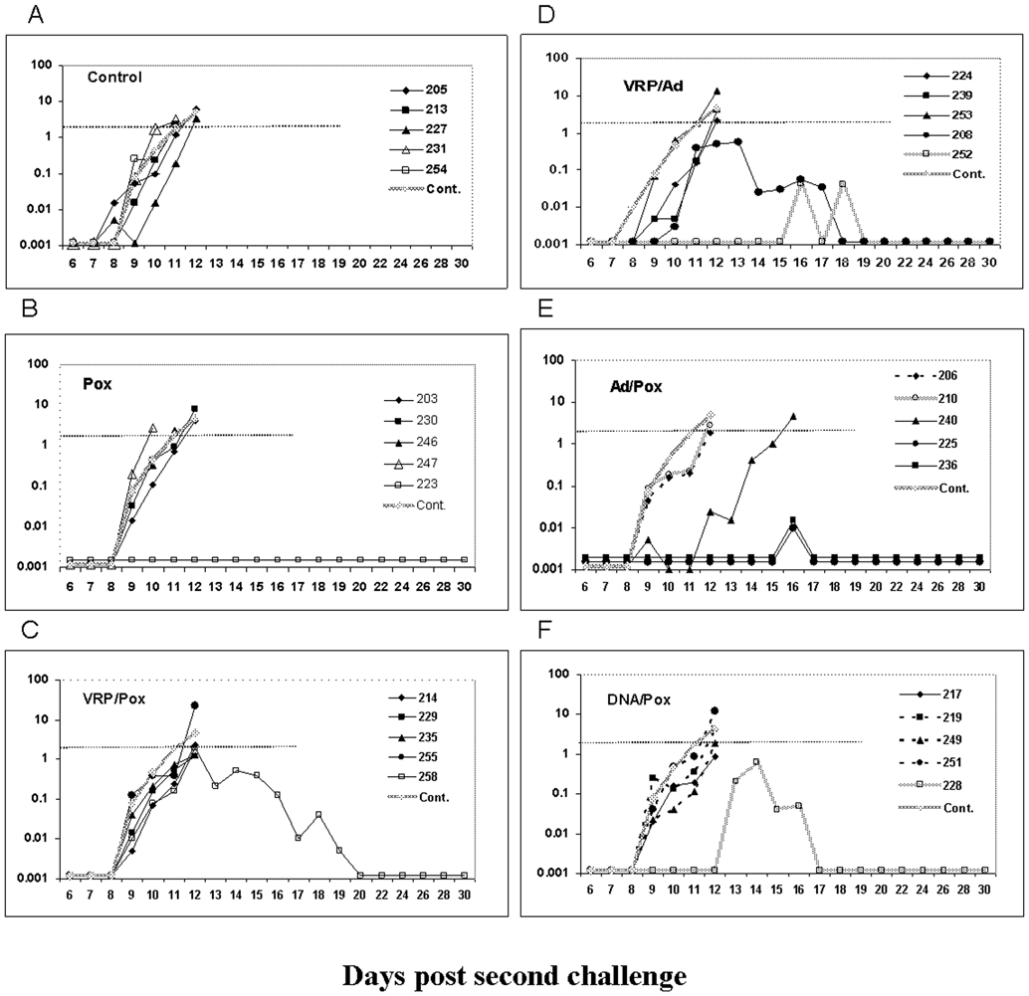
Daily parasitemias from individual monkeys after the second sporozoite challenge. Panel A, Control group: average parasitemia levels of 5 individual animals was presented as a thick grey line (Cont.) and is included in all 6 panels for comparison; Panel B, Pox group; Panel C, VRP/Pox group; Panel D, VRP/Ad group; Panel E, Ad/Pox group; Panel F, DNA/Pox group; The dotted line in each panel shows the 2% parasitemia level at which we treated animals with anti-malaria drugs. One monkey (223) in Panel C had no detectable parasitemia is shown as a horizontal line. The four monkeys which had no detectable parasitemias after the first challenge all developed parasites in the second challenge and are graphed with black interrupted lines. The three monkeys which self-cured their parasitemias after the first challenge are graphed with thick stippled lines.

Four months after the first sporozoite challenge, all animals received a second challenge with 100 P. knowlesi sporozoites given IV. Figure 5 shows the daily parasitemias for each monkey during the second challenge. All five Control monkeys became parasitemic at a mean 8.4 days after challenge, and all required drug treatment at mean day 11.4 (Figure 5 panel A). The four monkeys that were sterilely protected in the first challenge became parasitemic in the second challenge on day 9 and were treated on day 12 (Figure 5 panels E and F). Thus the vaccine responses that protected animals in the first challenge were not maintained long enough to protect them against the second challenge. Of the three monkeys which self-cured in the first challenge, two self-cured after the second challenge (Figure 5 panel D and E) and one required drug treatment (Figure 5 panel E). One monkey in the Pox group (223) was protected in the second challenge but not in the first (Figure 5 panel B). Protection of a monkey only in the second challenge but not in the first might seem paradoxical. We believe that this monkey did receive an adequate infectious challenge in the second round, as there were no technical problems with the injection. We believe that the most likely explanation is that the first challenge exposed monkeys to malaria parasites with multiple antigens that boosted vaccine induced immune responses. The complication of exposure to parasites after the first challenge makes further interpretation of protection data from the second challenge difficult.

## Discussion

The goal of this study was to improve upon the Pk4 DNA/Pox vaccine by replacing the DNA components with VRP or Ad5 vaccines. Unfortunately, while both of these novel vaccines were able to prime immune responses for boosting neither gave as much protection as priming with DNA plasmids.

Vaccination with DNA plasmids is potent in mice but much less effective in primates and humans where very large amounts of DNA are required to induce small immune responses. In mouse malaria vaccine studies using PyCSP antigen, VRPs and DNA have been comparable in priming responses for boosting with recombinant adenovirus or poxvirus (Doolan, personal communication). Unfortunately, in the present experiment VRPs were minimally immunogenic in themselves, and did not prime for protection with a poxvirus boost as well as did DNA. Although VRPs were the least effective priming modality we tested, a comparison with the Pox alone group shows that VRP priming did enhance protection and immunogenicity.

Recombinant Pk4 adenovirus type 5 vaccines were given to two groups of monkeys in these experiments: in the Ad/Pox group they were the prime and in the VRP/Ad group they were the boost. Comparing immune responses after priming alone (Fig. 4a and b), we are impressed that after a single priming dose, the monkeys receiving the Ad5 vaccines were able to mount antibody and T cell responses to most of the Pk4 antigens. We suspect that the one month interval between prime and boost for the Ad/Pox group was not optimal, and that a longer interval might lead to even better immunogenicity and protection.

Comparing the VRP/Pox and VRP/Ad groups allows us to assess the value of the Ad5 virus as a booster vaccine. Protection was at least as good if not better in the VRP/Ad group than the VRP/Pox group, and immune responses were equivalent. Thus we believe that the Ad5 provided a boost as potent as the poxviruses

The most striking finding of this study is the high level of sterile protection in the monkeys receiving the Pk4 DNA prime/poxvirus boost vaccine in the first challenge. Three of five monkeys (60%) never developed parasitemia after sporozoite challenge, and of the two monkeys that did become infected, one cured its parasitemia without the need for drug treatment. The fact that this protection was achieved using a DNA/poxvirus vaccine regimen suitable for humans is especially encouraging. In our previous four challenge experiments (Table 4), a total of 3/30 monkeys were sterilely protected by the Pk4 DNA/Pox vaccine, with protection ranging from 0 to 18%. Comparing the present study with the pooled results of our previous studies yields an Odds Ratio of 0.074 (95% CI 0.008, 0.636). Thus it is not likely that the improved protection is a random fluctuation due to the small number of experimental animals. Our hypothesis is that the increased protection may be due in part to the long intervals between vaccine doses used in this study, as has been seen in rodent malaria vaccine studies [32].

**Table 4:** Summary of Sterile Protection in Five Pk4 DNA/Pox Vaccine Studies.

Trial											N =	Sterile #	Sterile %
**a**	**DNA1**	**DNA2**		**DNA3**						**Pox**	**5**	**3**	**60**
	**day 0**	**28**		**96**						**420**			
													
**b**	**DNA1**	**DNA2**	**DNA3**					**DNA4**	**Pox**		**11**	**2**	**18**
	**day 0**	**30**	**60**					**280**	**310**				
													
**c**	**DNA1**	**DNA2**	**DNA3**				**Pox**				**5**	**0**	**0**
	**day 0**	**30**	**60**				**207**						
													
**d**	**DNA1**	**DNA2**	**DNA3**			**Pox**					**10**	**1**	**10**
	**day 0**	**30**	**60**			**156**							
													
**e**	**DNA1**	**DNA2**	**DNA3**		**Pox**						**4**	**0**	**0**
	**day 0**	**30**	**60**		**108**								

Summary of five published vaccine studies in rhesus monkeys using the Pk4 DNA/Pox vaccine and challenge with 100 Pk sporozoites IV. Trial a is the present experiment. Trial b is from Rogers (18). Trials c and e are from experiment 3 in Weiss (19), Trial d is from experiments 1 and 2 in Weiss (19). N gives the number of animals receiving the Pk4 DNA/Pox vaccine, and Sterile gives the number of animals which did not develop parasites in the blood. Longer regimens give higher proportion of sterilely protected animals.

However there are several caveats to be considered when comparing the present study with our previously published results. Because we have been working over a period of years, different production batches of plasmid and viral vaccines have been used. There are also some differences in the vaccine regimens, with the present study spreading the three priming DNA vaccinations over 4 months, and the study from Rogers et al. [18] including a fourth DNA dose before poxvirus boosting. Also, although challenge has always been with 100 P. knowlesi sporozoites the infectivity of those sporozoites no doubt varied between experiments. Finally, the rhesus monkeys in our studies have been obtained from several sources. In our previous studies using the Pk4 vaccine, we have used rhesus monkeys from breeding colonies founded with rhesus monkeys of Indian origin. In this study, we used rhesus monkeys of Chinese origin because Indian origin rhesus were not available at our institution. Although the immune responses to vaccines of genetic subgroups of rhesus monkeys may differ [33], in the absence of an immune correlate of protection we cannot know if genetic differences are responsible for the improved protection seen in this experiment. Because of all these concerns, the concept of longer vaccinations leading to better protection remains a conjecture which must be directly tested in a future experiment.

We were not able to identify an immune correlate of protection in this study. The two vaccines, DNA/Pox and Ad/Pox, which induced the most consistent immune responses to the P. knowlesi CSP were also the only two vaccines which sterilely protected monkeys (Table 2). From this, one might expect that the blood of protected monkeys would have higher antibody or T cell responses to CSP than non-protected monkeys. However, this was not the case (Table 3). One explanation for this seeming contradiction is that immune responses in the blood do not reflect protective immune responses in tissues. In mice, it has been shown that the immune responses that correlate with pre-erythrocytic malaria immunity occur within the liver tissue itself [34]. We think it likely that similar tissue specific liver immunity is occurring with pre-erythrocytic immunity in primates and humans as well, and that these immune events may not be easy to detect in the peripheral blood. We are undertaking studies of immune responses in the monkey liver to examine this concept.

Using flow cytometry, we were able to measure antigen specific responses from CD4+ T cells but we did not detect antigen specific CD8+ T cell responses. This is consistent with our previous studies of the Pk4 DNA/Pox vaccine [19], [28]. We had hoped that the VRPs or Ad5 viruses would be able to induce CD8+ T cell responses but this was not the case. CD8+ T cells are important immune effectors against liver stages of malaria in mice [35]–[37] and monkeys (Weiss, unpublished data) protectively immunized with radiation-attenuated malaria sporozoites. We believe that a vaccine which induces CD8+ T cell effectors may have increased efficacy against malaria liver stages.

The biggest failing of the P. knowlesi vaccines has been the short duration of protection: no sterilely protected animals in the first sporozoite challenge were sterilely protected in the second challenge four months later. This has also been the case in all of our previous studies. Lacking an immune correlate of protection, our vaccine development strategy is to improve the magnitude and longevity of all immune responses to malaria vaccine antigens, and to induce CD8+ effector T cells. Our next plan is to replace DNA priming with recombinant malaria proteins in novel adjuvants [38], [39]. We hope these next generation priming vaccines will allow stronger and longer lasting immune responses after boosting with recombinant viral vaccines, and a corresponding lengthening of vaccine efficacy.
